# Solute carrier nutrient transporters in rheumatoid arthritis fibroblast-like synoviocytes

**DOI:** 10.3389/fimmu.2022.984408

**Published:** 2022-10-20

**Authors:** Alyssa Torres, Brian Pedersen, Monica Guma

**Affiliations:** ^1^ Division of Rheumatology, Allergy and Immunology and School of Medicine, University of California, San Diego, San Diego, CA, United States; ^2^ Department of Medicine, Veterans’ Affairs (VA) San Diego Healthcare System, San Diego, CA, United States; ^3^ Department of Medicine, Autonomous University of Barcelona, Barcelona, Spain

**Keywords:** SLC transporters, FLS, RA, metabolism, nutrients

## Abstract

Metabolomic studies show that rheumatoid arthritis (RA) is associated with metabolic disruption. Metabolic changes in fibroblast-like synoviocytes (FLS) likely contribute to FLS abnormal response and strongly contribute to joint destruction. These changes often involve increased expression of nutrient transporters to meet a high demand for energy or biomolecules. The solute carrier (SLC) transporter families are nutrient transporters and serve as ‘metabolic gates’ for cells by mediating the transport of several different nutrients such as glucose, amino acids, vitamins, neurotransmitters, and inorganic/metal ions. In RA FLS SLC-mediated transmembrane transport was one pathway associated with different epigenetic landscape between RA and osteoarthritis (OA) FLS. These highlight that transporters from the SLC family offer unique targets for further research and offer the promise of future therapeutic targets for RA.

## Introduction

Rheumatoid arthritis (RA) is a complex autoimmune disease that classically affects the small joints of the hands and feet. Its pathogenesis is driven by an interplay between cells of the immune system and the cells comprising the tissues of a joint (1, 2). This includes T cells, B cells, macrophages, chondrocytes and fibroblast-like synoviocytes (FLS). The FLS in a joint affected by RA, are a key driver of the inflammatory response that leads to damage and loss of a structural integrity of the affected joint (3, 4). These FLS play a role in the dysregulation of the innate and adaptive immune response and increasing evidence demonstrate the multifaceted ways in which metabolic changes are involved in the pathogenesis of RA and abnormal RA FLS behaviour (5–7).

The SLC (solute carrier) gene superfamily current consists of 458 protein-coding genes with 65 distinct gene families (8). Genes within a family are grouped based only on the primary amino acid sequence. By definition, members of a family have >20-25% DNA sequence homology to one another. *In vitro* studies and animal models have demonstrated key functions of these transporters for basic nutrients and ions, energy metabolism, neurotransmission, and xenobiotic/drug transport (8, 9).

Not unexpectedly, SLC transporters are important in normal health and disease states (9, 10) This is evidenced by greater than half of SLC transporters being associated with either susceptibility to a human disease or directly attributable to a Mendelian human disease (11). This includes diseases affecting the immune system, cardiovascular disease, metabolic disease, neurological functioning and cancer. In comparison, ~20% of genes in general are known to be associated with human disease.

**Figure 1:**
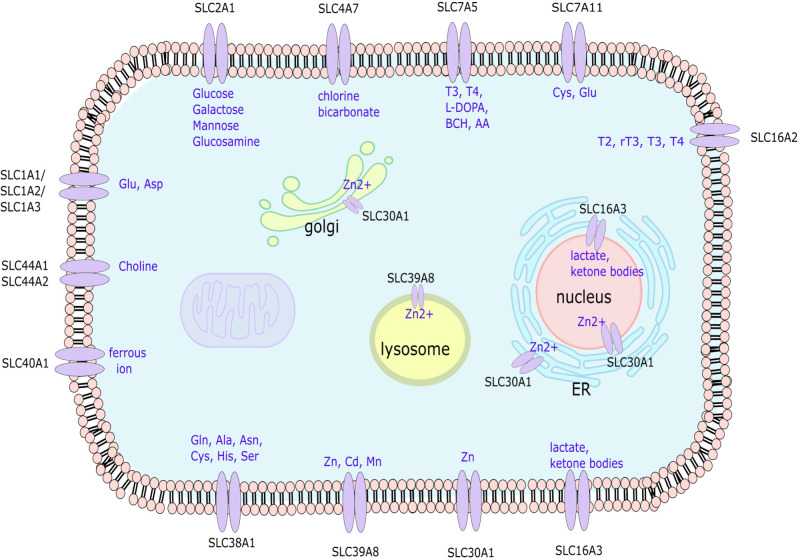
Depiction of the localization and solutes transported by the SLC transporter genes associated with RA FLS aggressive phenotype. SLC, solute carrier; Glu, glutamate; Asp, aspartate; Ala, alanine; Asn, asparagine; Cys, cysteine; His, histidine; Ser, Serine; Zn, zinc; Cd, Cadmium; Mn, Magnesium; T3, Triiodothyronine; T4, Thyroxine; L-DOPA, L-Dopamine; AA, amino acids; BCH, 2-aminobicyclo-(2, 2, 1)-heptane-2-carboxylic acid.

We recently described that in RA FLS, changes in the epigenetic landscape of genes are related to nutrient transporters, revealing a potential role of the SLC family in RA pathogenesis (12). In addition, several papers described associations of SLC transporter polymorphisms with RA (13–18). In this review, we concentrate on the roles of SLC transporters on the pathogenesis of RA. We focus our review on studies of SLCs in RA FLS as they are critical in regulating cell metabolism and are involved in the pathogenesis of RA (**Figure 1**).

## Importance of metabolic regulation on RA pathogenesis and SLC roles in metabolism regulation

Several studies have been conducted on the role of metabolism in RA pathogenesis. Different cell types in the inflamed synovium require various metabolites in order to meet energy demands including lactate, citrate, and succinate (5, 19, 20). This metabolic change is also accompanied by differences in immunity and inflammation and affects both stromal and immune cells (21). The result of these changes has also been described and explored by different groups and involves such pathways as glycolysis, pentose phosphate pathway, and biosynthetic activity (22). It is suggested that rewiring of the RA synovial membrane could modulate disease activity as has been shown in tumor cells and cancer (23). Some studies on metabolism and RA have focused on RA FLS in this inflammatory state, including specific pathways such as glucose metabolism, phospholipid metabolism, and bioactive lipids which differ from healthy synovial fibroblasts (24).

SLC transporters have been shown to be important in different physiological functions, but have also been studied for the effect of their expression on metabolite concentration in cells, and their involvement in various diseases (25). Some of these roles SLC transporters have on disease activity are effects on signaling, differentiation, and cell function by manipulating metabolite concentrations from normal levels, as seen in immune cells (26). For these reasons, SLC transporters have been explored as treatment targets for some conditions and diseases (27).

## SLC1 glutamate and neutral amino acid transporter family

The plasma membrane transporters for the neurotransmitter glutamate belong to the solute carrier 1 (SLC1) family (28). Other common names of these transporters include Excitatory Amino-Acid Carrier 1 (EAAC1 or SLC1A1), Glutamate Transporter (GLT1 or SLC1A2), Glutamate Aspartate Transporter 1 (GLAST or SLC1A3), Excitatory Amino acid Transporter 4 (EAAT4 or SLC1A6), and Excitatory Amino Acid Transporter 5 (EAAT5 or SLC1A7), to name a few. Glutamate transporters are of particular importance in the brain, where they contribute to the termination of excitatory neurotransmission, but they are also expressed in many tissues.

Constitutive expression of EAAC1 (SLC1A1), GLAST (SLC1A3), and GLT1 (SLC1A2) mRNA was shown in synovial tissue and confirmed by immunohistochemical (IHC) analysis (29, 30). Levels of glutamate increased in arthritis paws in a rat animal model of arthritis. When testing the effect of glutamate on increased proliferation positively correlated with higher concentrations of glutamate for RA rat fibroblast in comparison to normal rat fibroblasts. When given the non-selective EAAT inhibitors L-threo-B-hydroxyaspartate and carboxycyclopropyl)glycine (CCG-III), but nor GLT1 inhibitors, decrease glutamate accumulation and proliferation was significantly decreased despite higher levels of glutamate (31). These results imply a relatively selective dependence upon EAAT transporters to deliver glutamate for the metabolic needs of proliferating RA fibroblasts.

## SLC2 glut transporter family

All fourteen members of the SLC2 family encode GLUT proteins. The SLC2 or GLUT family are uniporters of hexoses,polyols and yet to be determined substrates and mediate the first step for cellular glucose usage (32). Deregulated glucose metabolism of tumor and non-tumor cells is known to affect cell behavior. RA FLS were found to have more glycolysis activity than OA FLS by our group and others (33, 34). SLC2A1 (also known as GLUT1) typically plays a role in basal glucose uptake. Interestingly, GLUT1 increased mRNA expression of RA FLS specifically correlated with increased glycolysis. Additionally, inhibitors of glycolysis, like 2DG and bromopyruvate, or glucose deprivation decreases proliferation and migration of RA FLS. The expression of GLUT1 mRNA in the synovial lining has been confirmed in a mouse model of inflammatory arthritis and glycolysis inhibition through BrPa (3-Bromo pyruvate) decreased arthritis severity in this model (33). Overexpression of hexokinase 2, which is specifically expressed in RA synovial lining and regulates FLS aggressive functions, also increased GLUT1 mRNA levels (35). Increased GLUT-1 and decreased GLUT-4 (SLC2A4) expression in RA synovium compared to OA synovium was confirmed for other groups (36). Of interest, TNF stimulation enhanced GLUT1 expression in RA FLS (37).

Another study looked at the effect of D-lactate and its relationship with GLUT1. The physiological concentration of lactate in normal tissues is about 1.5–3 mmol/L but can increase to 10–12 mmol/L at sites of inflammation such as rheumatic synovial fluid. When RA FLS were stimulated with D-lactate for six hours, GLUT1 mRNA, amongst other genes, was significantly increased through HIF1a, PI3K/Akt and NF-κB signaling pathways (38). Finally, another group studied the involvement of T lymphocytes in RA FLS by giving T cell conditioned media to RA FLS or culturing the FLS with CD4 T cells (39). CD4 conditioned media caused increased glycolysis in FLS and downregulated oxidative phosphorylation which was paralleled by increased both GLUT1 and GLUT3 mRNA. The above studies suggest that targeted changes in the activity of SLC2 family members could be exploited as adjunct therapeutic targets for RA.

## SLC4 bicarbonate transporter family

The SLC4 family members transport bicarbonate and consist of 10 genes that play roles in acid-base homeostasis (40). In addition to regulating pH, bicarbonate transporters contributes to a cell’s ability to fine tune the intracellular regulation of the cotransported/exchanged ion(s) (e.g., Na^+^ or Cl^−^) (41). In the context of these transporters playing a known role in migration of other cell types, one group has investigated the role of bicarbonate transporters on RA FLS migrations. Upon testing various genes of the SLC4 family, SLC4A7 (also known as NBCn1) was shown to be significantly increased in RA FLS upon TNF-a expression as compared to OA FLS (42). As a result, TNF stimulation significantly increased migration. NBCn1 was also found to be in the plasma membrane after TNF stimulation. Upon the use of NBC inhibitor S0859, RA FLS migration was attenuated. As bicarbonate affects pH and RA patients tend to have more acidic synovial fluid, they tested the effect of pH on FLS migration and found that more acidic conditions increased RA-FLS migration and NBCn1 expression. An NBC inhibitor, S0859, decreased RA FLS migration and reduce bone erosion and edema in joints in the collagen induced arthritis (CIA) model (42).

Similar to the findings of M. Ji et. al., our group also found SLC4A4 (NBCe1) expression to be increased in OA vs RA FLS (12, 42). Upon siRNA knockdown of SLC4A4, RA FLS were found to be more invasive but with unchanged migration compared to control siRNA (12). This collection of findings demonstrates the potential direct role of the SLC4 bicarbonate transporters in regulating the pH of pathologic synovium and their role in RA disease progression.

## SLC7 cationic amino acid transporter/glycoprotein associated family

The SLC7 family transports amino acids, particularly, SLC7A5 also known as LAT1, transports large, neutral, L-type amino acids (43). Amino acids play essential roles in cell biology as regulators of metabolic pathways. In RA FLS, the heterodimer of SLC3A2 and SLC7A5 mediates cellular uptake of large neutral amino acids like phenylalanine (39), tyrosine, leucine, and tryptophan (28). SLC7A5 is overexpressed in RA synovial tissue as compared with OA synovial tissue, and found to colocalize with FLS through immunofluorescence of synovial tissue from RA patients (44). This group also found that IL1β stimulation upregulated SLC7A5 and induced mTORC1 activation. Another group utilized RNAi and the LAT1 inhibitor, L-leucine analogue b(−)2-aminobicyclo[2,2, 1]heptane-2-carbocyclic acid (BCH), to test the effect on downstream mechanisms (45). Both methods resulted in reduced phosphorylation of mTOR and 4EBP1, leucine uptake and migration in RA FLS. Interestingly, IL-17 increased expression of SLC7A5, which was attenuated by inhibiting the mTOR pathway (45).

SLC7A11 is a transporter that is targeted by the RA drug sulfasalazine and is also referred to as xCT or system XC-. This transporter specifically exports glutamate and imports cysteine. Cysteine is essential for glutathione synthesis which has effects in ferroptosis and reactive oxygen species resistance (46). One study investigated the effect of icariin (47), shown to promote anticancer, antiaging, neuroprotective, and anti-inflammatory effects, on ferroptosis in FLS (48). FLS stimulated by lipopolysaccharide (LPS) were used as a synovitis cell model. ICA inhibited cell death, increased cell viability, reduced lipid peroxidation, reduced iron content, and inhibited ferroptosis-related proteins as compared to controls. ICA inhibited ferroptosis by activating the XC-system and glutathione peroxidase 4 (GPX4) activity, suggesting a role of ferroptosis in FLS aggressive phenotype.

Of interest, a recent study used an opposing approach and used imidazole ketone erastin (49), which is a ferroptosis inducer, to decrease fibroblast numbers in synovium accompanied by a TNF antagonist, to halt progression of arthritis in the collagen-induced arthritis (CIA) mouse model. This study found that ferroptosis resistance in FLS was regulated by TNF by upregulating SLC7A11, glutamate-cysteine ligase catalytic subunit (GCLM), and glutamate-cysteine ligase regulatory subunit (GCLC) and increasing cysteine uptake. A TNF antagonist allowed RA FLS to become more susceptible to ferroptosis, so the combination of a TNF antagonist and a ferroptosis inducer had a synergistic effect in RA FLS but not in healthy synoviocytes (50). A similar approach also described that ferroptosis is decreased in RA FLS by looking at mitochondrial morphology and membrane potential in RA FLS along with increases in SLC7A11 (51). They also looked at the effect of glycine on ferroptosis (51) Glycine enhanced ferroptosis by increasing GPX4 promoter methylation, which decrease dGPX4 expression, and by increasing the expression of ferritin heavy chain 1 (FTH1), which releases iron and induces ferroptosis. In addition, glycine reduced progression of ferroptosis in the CIA mouse model. The above studies suggest that the modulation of ferroptosis, as shown with sulfasalazine (52), by targeting SLC7A11 among other strategies can be exploited as adjunct therapeutic targets for RA.

## SLC16 monocarboxylate transporter family

The SLC16 family (specifically SLC16A1, SLC16A3, SLC16A7, and SLC16A8) transports monocarboxylates such as l-lactate, pyruvate, and ketone bodies across the plasma membrane (53). SLC16A2 is specifically involved in transporting thyroid hormones, while the other nine transporters are involved in transporting other molecules or their role is otherwise known.

SLC16A3, also known as monocarboxylate transporter 4 (MCT4), is known to transport lactate and ketone bodies. SLC16A3 mRNA and protein expression is upregulated in RA compared to OA FLS and exports intracellular lactate into the extracellular space (54). MCT4 siRNA reduced RA FLS proliferation in comparison to OA FLS. This effect was mediated by increased apoptosis. In an *in vivo* mouse model of collagen induced arthritis (CIA), MCT4 siRNA was electroporated into the joint tissue of mice, decreased MCT4 levels and was associated with reduced synovial cell hyperplasia and infiltration. However, no difference in severity of cartilage destruction and bone erosion was observed.

## SLC30 zinc efflux family and SLC39 metal ion transporter family

Zinc ions are essential in many physiological processes, including enzyme catalysis, protein structural stabilization, and the regulation of many proteins, including matrix metalloproteinases (MMPs), a disintegrin and metalloproteinase (ADAM), and a disintegrin and metalloproteinase with thrombospondin motif (ADAMTS) (55, 56). These proteases play key roles in the formation, homeostasis and remodeling of the extracellular matrix and the cartilage. The SLC30 family are specifically *exporters* of zinc and are commonly referred to as Zinc transporters (ZnTs). In contrast, the SLC39 family are *importers* of zinc and are commonly referred to as Zrt- and Irt-like proteins (ZIPs), but can also transport a handful of other metals. In FLS, it has been found that upon TNF and IL-17 stimulations, zinc exporters (SLC30) and importers (SLC39), there are variable effects on Zinc exporters (SLC30) and importers. With this cytokine combination, SLC39A8 (also known as ZIP-8) expression significantly increased in RA FLS. However, in OA FLS, only TNF was able to increase ZIP-8 expression, and no difference was observed when IL-17 was added. With increased amounts of Zinc, ZIP-8 expression was inhibited in RA FLS after TNF and IL-17 stimulation, but not in OA FLS. In contrast, SLC30A1 (also known as ZnT1) expression was unchanged upon stimulation with TNF and IL-17, and expression was slightly enhanced with increased amounts of zinc, but upon the combination of zinc and cytokines, ZnT1 expression was significantly increased. This combination of cytokines and zinc also significantly increased IL-6 production in RA FLS (57).

## SLC38 system A and system N sodium-coupled neutral amino acid transporter family

SLC38 family is a set of genes that primarily transports amino acids. Specifically, SLC38A1, or system N amino acid transporter 1 (SNAT1), is a sodium coupled amino acid transporter that is shown to be upregulated in human liver cancer cell lines (58). SLC38A1 was shown to have differential epigenetic markings between RA and OA FLS with higher expression in RA FLS than in OA FLS. Upon siRNA knockdown, there was no difference in migration or invasion, suggesting a s redundancy in amino acid transporters (12).

## SLC40 basolateral iron transporter family

The SLC40 family consists of only one SLC named SLC40A1, which is also called MTP1 or IREG1, and codes for a protein named ferroportin-1 (FPN1). It is involved in the cellular efflux of iron (59). In the paper previously described for SLC7A11 (50), this group briefly analyzed expression of SLC40A1 and its role in RA FLS ferroptosis. In this model, RA FLS incubated with a TNF antagonist were more sensitive to ferroptosis upon sensitization with IL-6. They found that IL-6 decreased expression of SLC40A1 and lowered ferritin. Upon knockdown of SLC40A1, RSL3-driven ferroptosis toxicity was increased. Based on these findings, IL-6 caused an increase in intracellular iron levels which could contribute to RA FLS proliferation. Of note, increased intracellular iron levels positive correlates with tumor growth (33).

## SLC44 choline-like transporter family

The SLC44 family comprises several high affinity choline carriers which are thought to provide choline for phospholipid and acetylcholine synthesis (60). Choline kinase (ChoKα), an essential enzyme for phosphatidylcholine biosynthesis, is required for cell proliferation and has been implicated in cancer invasiveness. The relevance of choline, choline kinase and phosphocholine in RA FLS aggressive phenotype and macrophage activation was described by our group and others (31, 61, 62). SLC44A1 (also known as CTL1) is expressed widely in the nervous system while the others, including SLC44A2 (also known as CTL2) are expressed in peripheral tissues. SLC44A2 is thought to be involved in autoimmune hearing loss

One group found all five of the CTL proteins present in synovial tissue and cartilage of human joints. CTL1 and 2 being the most prominently expressed, but without a difference between OA and RA FLS (63). CTL1 and CTL2 were found to localize to both macrophages and fibroblasts in immunohistochemical staining, with CTL1 being the most prominent in synovium and cartilage (64). Another study found CTL1 and CTL2 mRNA and protein to be highly expressed in RA FLS and localized to the plasma membrane. They found that CHT1, CTL4, CTL5, OCT1, and OCT2 mRNAs were not expressed in RA FLS. CTL3 and OCT3 were expressed at low levels and proposed that inhibition of these transporters promote apoptotic cell death (65). In RA FLS, the exposure to the choline uptake inhibitor hemicholinium-3 (HC-3) in dose specific manner, decreased cell viability and increased caspase 3/7 activity; both of which are markers of apoptosis (65).

## SLC47 multidrug and toxin extrusion family

The SLC47 family is called the multidrug and toxin extrusion (MATE) family which comprises of SLC47A1 (or MATE1) and SLC47A2 (or MATE2). MATE1 and MATE2 are H+/organic cation antiporters that secrete organic cations and are mostly expressed kidney and liver (66).

One study looked at the effect of tyrosine kinase inhibitors (TKIs) in RA FLS and the role of these membrane transporters (67). The tyrosine kinase inhibitor Imatinib was studied for transport and accumulation and found that MATE1 had the greatest affinity for Imatinib. This was confirmed after siRNA knockdown of MATE1 caused decreased uptake of Imatinib transport into RA FLS With increased activity of MATE1 following PDGF stimulation, proliferation was inhibited by imatinib given its ability to enter the cell. When cells were under disease relevant conditions such as a lower pH and cytokine stimulation, imatinib uptake decreased. Previous studies suggested a potentiation of the effect of imatinib under these disease relevant conditions.

A different study found MATE1 to be increased in OA as opposed to RA. MATE1 was also determined to mediate transport of Tofacitinib, a blocker of the ATP-binding side of Janus kinase (JAK) proteins and tyrosine kinase inhibitor, from RA FLS (68). The increased expression of MATE1 in OA allows tofacitinib to be exported out of the cell, while the lower activity of MATE1 in RA does not allow tofacitinib to leave the cell, thus allowing tofacitinib to undergo its intended effects. It is suggested that tofacitinib is a more ideal drug since healthy cells would be able to export it through MATE1 and thus not undergo JAK inhibition while RA FLS would not be able to export it due to lower MATE1 expression.

## The role of SLC transporters in other cells

SLC transporters in RA are relevant in more than just FLS. Other cell types critical in RA pathogenesis, including chondrocytes, CD4+ T cells, monocytes, endothelial cells and neutrophils express SLC transporters and they play a role in their activated phenotype. **Table 1** summarizes they key findings.

**Table 1:** The role of SLC transporters in RA cells other than FLS.

SLC family	Gene	Alias	Substrates	Cell type	Findings	Reference
SLC2	SLC2A1 SLC2A2	GLUT1 GLUT2	DHA, glucose, galactose, mannose, glucosamine	Chondrocytes	DHA transported *via* GLUT1 regulated by hypoxia	(69)
SLC5	SLC5A12	SMCT2	Short chain fatty acids	CD4+ T cells	Lactate regulates T-cell migration and regulates metabolism and inflammation	(70), (47)
SLC7	SLC7A5	LAT1	Large neutral AA	Monocytes Macrophages	Glycolytic reprogramming through SLC7A5 in immune response	(71)
SLC7	SLC7A11	XC-	Cysteine, glutamate	Endothelial cells	Sulfasalazine inhibits fibroblast growth-factor induced chemotaxis of endothelial cells	(72)
SLC16	SLC16A1	MCT1	Lactate, pyruvate, ketone bodies	T cells	Lactate regulates T-cell migration and regulates metabolism and inflammation	(70)
SLC29	SLC29A1	ENT1	Nucleoside	T cells in animal model	Decitabine therapy results in long-term remission of RA	(72)
SLC29	SLC29A1 SLC29A2	ENT1 ENT2	Nucleoside	Mixed synovial cells	Dipyridamole is an ENT inhibitor. It did not affect cytokine release in RA and OA cells	(73)
SLC38	SLC38A1 SLC38A2 SLC38A3	SNAT1 SNAT2 SNAT4	Gln, Ala, Asp, Cys, His, Ser	Monocytes Neutrophils AIA	Glutamine uptake is regulated by SNAT and attenuated AIA	(74)
SLC39	SLC39A8	ZIP8	Zinc	Macrophages	Zinc regulated IL-1β	(75)
SLC63	SLC63A2	SPNS2	Sphingolipids	CIA model	SPNS2 deletion improved arthritis	(76)

CIA, collagen induced arthritis; AIA, antigen induced arthritis, AA, amino acids; GLUT, glucose transporter; SMCT2, sodium-coupled monocarboxylate transporters; LAT1, L-type amino acid transporter; MCT1, monocarboxylate transporter 1; ENT, Equilibrative nucleoside transporter; SNAT, Sodium-Coupled Neutral Amino Acid Transporter; ZIP, Zrt- and Irt-like proteins; SPNS2, Sphingolipid Transporter 2; DHA, Dehydroascorbate

## SLC transporters as therapeutic targets

The SLC family offer unique targets for further research and offer the promise of future therapeutic targets for RA. Importantly, many SLC transporters are expressed on the cell surface and are therefore targetable by both small molecules and therapeutic antibodies. Modulation of these SLC can also affect both the efficacy and toxicity of RA therapies as suggested by the association of polymorphism with methotrexate toxicity (77–82). A comprehensive understanding of the function of these transporters and the pathways in which they act will be critical for recognizing SLC that may be therapeutically targeted in RA.

## Author contributions

AT, BP, MG have contributed to the literature review. All authors were involved in drafting the article or revising it critically for important intellectual content. All authors contributed to the article and approved the submitted version.

## Funding

This work was supported by the National Institutes of Health (R01AR073324 to MG).

## Conflict of interest

The authors declare that the research was conducted in the absence of any commercial or financial relationships that could be construed as a potential conflict of interest.

## Publisher’s note

All claims expressed in this article are solely those of the authors and do not necessarily represent those of their affiliated organizations, or those of the publisher, the editors and the reviewers. Any product that may be evaluated in this article, or claim that may be made by its manufacturer, is not guaranteed or endorsed by the publisher.
